# Recent advances in the extraction, chemical composition, therapeutic potential, and delivery of cardamom phytochemicals

**DOI:** 10.3389/fnut.2022.1024820

**Published:** 2022-09-30

**Authors:** Abdullah, Naveed Ahmad, Wenni Tian, Song Zengliu, Yucheng Zou, Shahzad Farooq, Qingrong Huang, Jie Xiao

**Affiliations:** ^1^Guangdong Provincial Key Laboratory of Functional Food Active Substances, College of Food Sciences, South China Agricultural University, Guangzhou, China; ^2^Multan College of Food & Nutrition Sciences, Multan Medical & Dental College, Multan, Pakistan; ^3^College of Biosystems Engineering and Food Science, Zhejiang University, Hangzhou, China; ^4^Department of Food Science, Rutgers, The State University of New Jersey, New Brunswick, NJ, United States

**Keywords:** cardamom, phytochemicals, extraction techniques, bioactivities, encapsulation and delivery, bioavailability

## Abstract

Dietary phytochemicals including plant-derived alkaloids, carotenoids, organosulfur compounds, phenolics, and phytosterols, are health-promoting bioactive compounds that help in the prevention and mitigation of chronic diseases and microbial infections beyond basic nutrition supply. This article covers recent advances in the extraction, chemical composition, therapeutic potential (nutraceutical and antimicrobial), and delivery of black and green cardamom-derived phytochemicals. In recent years, advance extraction techniques (e.g., enzyme- assisted-, instant controlled pressure drop-, microwave- assisted-, pressurized liquid-, sub- critical-, supercritical fluid-, and ultrasound-assisted extractions) have been applied to obtain phytochemicals from cardamom. The bioactive constituents identification techniques, specifically GC-MS analysis revealed that 1,8-cineole and α-terpinyl acetate were the principle bioactive components in black and green cardamom. Regarding therapeutic potential, research findings have indicated desirable health properties of cardamom phytochemicals, including antioxidant-, anti-hypercholesterolemic, anti-platelet aggregation, anti-hypertensive, and gastro-protective effects. Moreover, antimicrobial investigations revealed that cardamom phytochemicals effectively inhibited growth of pathogenic microorganisms (bacteria and fungi), biofilm formation inhibition (Gram-negative and Gram-positive bacteria) and bacterial quorum sensing inhibition. Encapsulation and delivery vehicles, including microcapsules, nanoparticles, nanostructured lipid carriers, and nanoliposomes were effective strategies to enhance their stability, bioavailability and bioefficacy. In conclusion, cardamom phytochemicals had promising therapeutic potentials (antioxidant and antimicrobial) due to polyphenols, thus could be used as functional additive to increase shelf life, inhibit oxidative rancidity and confer pleasant aroma to commercial edibles as well as mitigate oxidative stress and lifestyle related chronic diseases (e.g., cardiovascular and gastrointestinal diseases). A future perspective concerning the fabrication of functional foods, nutraceuticals and antibiotics to promote cardamom phytochemicals applications as biotherapeutic agents at large-scale requires thorough investigations, e.g., optimum dose and physical form of supplementation to obtain maximum health benefits.

## Introduction

Cardiovascular disparities, foodborne pathogens and microbial recurrent infections have motivated the researchers to discover effective food-based biomedicines and antimicrobials to attenuate diseases and inhibit growth of pathogenic microorganisms. Various potent medicines are available to counter diseases and pathogens, nevertheless, side effects on human health are often associated with their applications. Therefore, physiological dysfunctions and foodborne diseases treatment through phytochemicals has gained tremendous research attention due to their safe nature along with nutritional perspectives, instead of pharmaceuticals. Applications of dietary phytochemicals in food, cosmetic, nutrition, and pharmaceutical industries have seen an emerging trend due to their health-enhancing traits such as antiradical, antimicrobial, cardio-protectant, and gastro-protective properties (1–4).

Dietary phytochemicals include plant-derived alkaloids such as caffeine, carotenoids (e.g., carotene, cryptoxanthin, lutein, and lycopene), organosulfur compounds (e.g., isothiocyanates, indoles, and sulforaphane), phenolics (e.g., coumarins, flavonoids, phenolic acids, stilbenes, and tannins), phytosterols (e.g., avenasterol, campesterol, campestanol, citrostadienol, stigmasterol, stigmastanol, sitosterol, and sitostanol), tocols (e.g., tocopherols and tocotrienols), fatty acids, and volatile compounds. These are health-promoting bioactive compounds that help in preventing and mitigating physiological disorders and risk of chronic infections. Such phytochemicals are rich sources of organic antioxidants and antimicrobials that possess health benefits, including boosting antioxidant enzymes (glutathione and superoxide dismutase), decreasing malondialdehyde contents, microbial communication and biofilm formation inhibition, and prevention and mitigation of various chronic diseases (1, 4–6).

Cardamom, a perennial herbaceous plant belongs to the family *Zingiberaceae*, and green cardamom (*Elettaria cardamomum*) and black cardamom (*Amomum subulatum*) are two major cultivars. These are grown in different regions of the world, including Bhutan, India, Mexico, Nepal, Peninsula, Sri Lanka, Tanzania, Thailand, and Vietnam. Regarding yearly production, India is the leading producer contributing 4000 metric tons followed by Nepal and Bhutan with 2500 metric tons and 1000 metric tons, respectively (2, 7, 8). To date, different extraction techniques including conventional extraction methods (e.g., hydrodistillation and Soxhlet apparatus) and advance procedures (e.g., ultrasound- assisted-, microwave- assisted-, supercritical fluid-, sub- critical-, pressurized liquid-, enzyme- assisted-, instant controlled pressure drop-, and solar energy-based extractions) have been used for the extraction of dietary phytochemicals. Among the various extraction techniques, supercritical fluid extraction (SFE) has the potential advantages of controlled operative conditions (time, temperature and pressure), less sample degradation, accelerated mass transfer, and no further sample clean-up (1, 6, 9, 10).

After phytochemicals extraction, various chromatographic techniques are being used to characterize and quantify the bioactive constituents. For example, different fractions of fatty acids and phytosterols, tocopherols, and lipids present in the sample can be identified by gas chromatography, high performance liquid chromatography and column chromatography, respectively (4). The chromatographic analysis of cardamom phytochemicals indicated α-terpinyl acetate and 1,8-cineole (potent antioxidant) were the major bioactive constituents in green and black cardamom, contributing pleasant and pungent aroma, respectively. In addition, various other bioactive constituents, including sabinene, linalool acetate, nerolidol, thujene, pinene, cymene, limonene, geranial, and myrcene were also present in cardamom (2, 3).

In recent years, the desirable health-promoting properties of cardamom phytochemicals have been reported, including antioxidative-, antimicrobial-, anti-ulcer (gastrointestinal protective), anti-hypercholesterolemic, anti-platelets aggregation, and anti-hypertensive activities (1, 6, 11). Nagashree et al. (12) reported that cardamom phytochemicals significantly decreased the levels of low-density lipoproteins, very low-density lipoproteins and triglycerides in rats nourished with a high-fat diet. Abdullah et al. (2) described that cardamom phytochemicals inhibited the growth of foodborne fungi (*Candida albicans*) and bacteria (*Bacillus cereus, Streptococcus mutans*, *Staphylococcus aureus*, and *Listeria monocytogenes*), biofilm formation in Gram-positive (*Salmonella* Typhimurium) and Gram-negative bacteria (*Escherichia coli*) and quorum sensing phenomena (*Chromobacterium violaceum*). Qiblawi et al. (13) indicated that cardamom substantially inhibited the aspirin-induced gastric lesions in rats, when different cardamom phytochemicals were applied such as methanolic extract (100-500 mg/kg) and essential oil (12.5-50 mg/kg).

**FIGURE 1:**
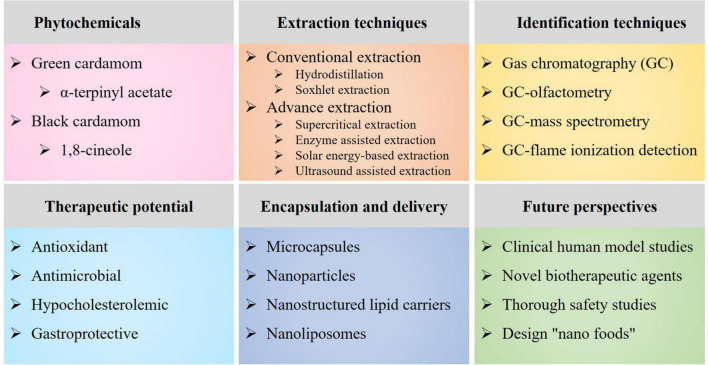
An overview diagram elaborating the main cardamom phytochemicals, extraction techniques, identification techniques, therapeutic potential, encapsulation and delivery, and future perspectives.

This article covers recent advances in the extraction, chemical composition, therapeutic perspectives, and encapsulation and delivery of cardamom phytochemicals (Figure 1). The various extraction techniques, including conventional- (e.g., hydrodistillation and Soxhlet extraction) and advance extraction techniques (e.g., enzyme- assisted-, instant controlled pressure drop-, microwave- assisted-, pressurized liquid-, solar energy- based-, sub- critical-, supercritical fluid-, and ultrasound-assisted extractions), and bioactive constituents identification techniques [e.g., gas chromatography (GC), GC-mass spectrometry (GC-MS) and GC-flame ionization detection (GC-FID)] were briefly summarized. Moreover, the health-promoting characteristics with a special emphasis on the anti- oxidative-, antimicrobial-, cardio-protectant and hypo- cholesterolemic-, gastro- protective-, and biosafety and non-mutagenic properties were discussed. In addition, future perspectives focusing nutrition-related disorders, foodborne pathogens and microbial infections were highlighted, i.e., dietary phytochemicals as a green alternative to chemical preservatives, pharmaceuticals and conventional antibiotics.

## Phytochemicals extraction techniques

To obtain phytochemicals, the extraction method, sample pre-treatments, sample pulverization, extraction solvent, and extraction time are important factors. The type of solvent (i.e., polar, non-polar, lipophilic, hydrophilic, liquid, volatile, non-volatile, solid, or gas) and particle size (pulverization degree) of sample material are critical factors. Extraction solvent must be selected based on extraction methods, especially, SFE- and enzyme-assisted extractions combining co-solvent like hexane and acetone is advantageous for combined non-polar and polar effects (14). For extracting polar bioactive molecules using SFE, a polar modulator (ethanol as co-solvent) may be introduced in a small amount (15). For example, hexane extract contained only 0.3% α-terpinyl acetate when compared to supercritical extract that contained 42.3%. The obvious reasons include organic solvent pollution, the presence of cuticular wax and thermal degradation of α-terpinyl acetate (16). The particle size is also considered a compelling factor in SFE to obtain higher yield of phytochemicals as the length of diffusion path may control the internal mass transfer mechanism (16). Size reduction or pulverization degree reduces or exploits the surface area that makes it a critical factor as the desired components within tissues and cells are exposed. An ultrasound sound extraction from almonds reported enhanced ultrasonic action with greater yield with greater pulverization degree (17). Similarly, Balachandran et al. (18) reported enhanced diffusivity and yield of gingerols with reduced particle size. Thus, pulverization degree is an important experimental parameter that greatly influences extraction yield (15, 19).

Various extraction procedures have been used to obtain phytochemicals, and can be classified as conventional- (e.g., hydrodistillation, steam distillation and Soxhlet extraction) and advance extraction methods (e.g., enzyme- assisted-, instant controlled pressure drop-, microwave- assisted-, pressurized liquid-, solar energy- based-, sub- critical-, supercritical fluid-, and ultrasound-assisted extractions) (2, 9, 10, 20–25). Conventional extraction methods have wider applicability in the extraction of phytochemicals due to ease of operation, however, they require more time and huge amount of solvents, and give less extraction yield, thus making up-scaling difficult. Importantly, the conventional methods are also not suitable for thermolabile phytochemicals due to their adverse extraction conditions. Therefore, advance extraction techniques, specifically, hybrid approach, i.e., combination of two extraction methods has been practiced due to their potential advantages of less extraction time, low amount of solvents consumption, higher extraction yield, and improved quality and physical stability of phytochemicals. Table 1 compares the conventional and advance extraction techniques in terms of extraction time, solvents consumption, instrumentation cost, sample size, and extraction yield and quality.

**TABLE 1:** A comparison between conventional and advance extraction methods being used for phytochemicals extraction.

			Parameters		
	
Extraction technique	Extraction time	Solvents consumption	Instrumentation cost	Sample size	Extraction yield/quality
**Conventional extraction**					
Cold press/Mechanical press	Relatively long	Not applicable	Out-dated (normal)	Usually large	Low/needs further processing
Distillation (HD/SD)	In hrs.	Fairly large	Low-Normal	Large	Low to comparable yield with inferior quality
Soxhlet/solvent	In hrs.	Fairly large	Low-Normal	Large (repeated many times)	Low to comparable yield with inferior quality
**Advance extraction**					
UAE	Very short (in minutes usually)	Not applicable	Costly	Small	Higher/superior
EAE	Very short (in minutes usually)	Small	Low to medium cost	Small	Higher/superior
MAE	Very short (in minutes usually)	Not applicable	Low to medium cost	Small	Higher/superior
SFE	Very short (in minutes usually)	Small	Costly	Small	Higher/superior

HD, hydrodistillation; SD, steam distillation; UAE, ultrasound-assisted extraction; EAE, enzyme-assisted extraction; MAE, microwave-assisted extraction; SFE, super-critical fluid extraction.

## Conventional extraction methods

Hydro- or steam distillation is still a widely used extraction method despite being conventional and using a large amount of solvent (water) due to wider applicability and ease of operation (21). In this method, extraction is based on the principal that combined vapor pressure equals the atmospheric pressure at their boiling temperatures. The steam is charged and encounters a narrow vessel chilled by an external source (cold water). Subsequently, the condensed steam is collected in a vessel with oil being less dense on the top (25, 26). An 8 h steam distillation was used to extract 3.5% volatile oil from black cardamom fruits (27). Oil from the fruits of two varieties of green cardamom from Thailand was also extracted by steam distillation (28). Noleau et al. (29) used a simultaneous distillation extraction apparatus for 1 h with dichloromethane to redistill volatiles, which resulted a yield of 3.68% for green cardamom capsules. Large cardamom seed oil was extracted by steam distillation with 2.50% yield (30). Black cardamom seeds (60% moisture, locally dried, and 5% moisture) presented 1.60, 2.70, and 2.40% hydrodistilled oil respectively (31). The black cardamom pericarp (husk) was also extracted for its oil contents by Clevenger hydrodistillation, which gave 0.18% yield (32). Kumar et al. (33) also isolated the oil from seeds and fruit coat of green cardamom from Indian region. They used 6 h hydrodistillation in Clevenger-type apparatus and 6.0 and 1.40% oil yields were obtained from seeds and fruit coat, respectively. Marongiu et al. (16) extracted cardamom seed oil by 4 h hydrodistillation with 5% yield in a Clevenger-type circulatory apparatus with 100 g material.

The black cardamom fruit was hydrodistilled for its oil contents using 500 g sample and all glass apparatus, which yielded 1.80% oil (34). Four predominantly used varieties of green cardamom from the Indian vicinity were sequentially extracted for their seed oils using solvents like hexane, methanol, water, dichloromethane, and ethyl acetate. The yield obtained by hydrodistillation varied from 7.90 to 8.79% (35). Black cardamom seed and rind (110 and 110 g pulverized sample) gave 4.50 and 1.00% yield when extracted using Likens-Nickerson hydrodistillation apparatus for 4 h with dichloromethane as an extracting solvent (36). The black cardamom was collected from six different altitudes and a 2 h hydrodistillation in all glass Clevenger-type apparatus having 20 g pulverized black cardamom seeds with 400 mL distilled water was used to extract their oil fraction. The yield varied from 0.98 to 1.95% (37). A 24 h hydrodistillation was carried out with 20 g sample (green cardamom seeds) and 500 mL of water which yielded approximately 66.43 mg extract/g sample (26). Jaramillo-Colorado et al. (38) extracted cardamom seed oil from three species by 2 h hydrodistillation with 500 g plant material and a 5 L-round flask and reported the presence of α-terpineol acetate and 1,8-cineol as predominant phytochemicals. Eikani et al. (22) used 10 g green cardamom sample (35 mesh size) to extract the oil using Clevenger-type hydrodistillation with 3.80% yield. The Iranian dried green cardamom sample (20 g) was completely submerged in water and hydrodistilled for 4 h in a Clevenger-type full glass apparatus with 3.10% yield (39). Cardamom seed powder was hydrodistilled for 5 h using 300 mL distilled water and 25 g sample size using Clevenger apparatus. Approximate yield was 45 mg extract/g dry seed (40). Morsy used 40 g cardamom seed powder (sieved thru 0.5 mm screen) to extract seed oil by hydrodistillation for 6 h by Clevenger-type apparatus oil according to the European Pharmacopoeia with approximate yield of 7.05% (41). The oil from dried aerial parts of green cardamom was hydrodistilled in all glass Clevenger-type apparatus for 3 h with 5.70% yield (42). Four different cultivars of black cardamom from Indian regions were used for oil extraction by hydrodistillation which presented 1.2–2.8% oil yield (43). Govindarajan et al. (44) used modified Clevenger-type hydrodistillation apparatus to extract oil (0.73% yield) from fresh leaves of black cardamom for 3 h. Noumi et al. (45) obtained green and black cardamom seed oils by hydrodistillation technique. Two varieties of Chinese black cardamom (CBC-*Amomum tsao-ko*) were compared for their oil composition from Indian green and black cardamom. All varieties were hydrodistilled for their oil fraction. Chinese black cardamom yield ranged from 0.7 to 1.8% while black cardamom yielded 0.9–1.5% (46). Wang et al. (47) also used hydrodistillation (5 h) to extract black cardamom oil with varying temperatures to optimize extraction yield. The maximum oil yield was 3.71% when black cardamom was dried at 55°C for 32 h. Sontakke et al. (48) obtained 2.6% extraction yield by steam distillation (5 h) using 150 g sample. A study reported cardamom essential oil extraction from 22 accessions in Clevenger apparatus (hydrodistillation) with a 3 h optimized time and average yield of 4.5–9.5% (three replicates, 20 g sample, 500 mL distillation flask) (49). Green cardamom was extracted for its seed oil by hydrodistillation with a yield of 2.52% (25). Despite few confines, hydrodistillation is among the most utilized extraction method among the conventional approaches.

Soxhlet (solvent) extraction also continued to be an option amongst conventional approaches: the Soxhlet assembly uses a sample thimble, heating mental, condenser, and a receiving flask. The most commonly used organic solvents are hexane, petroleum ether, alcohol, acetone, methanol, chloroform, and ethanol (40, 48, 50, 51). Cardamom seeds were extracted through solvent extraction for their oil contents using hexane by Marongiu et al. (16). The extraction was carried out using 12 g sample at 25°C for 24 h with 7.6% yield. For comparative purpose, as a control treatment, 45 g cardamom sample was extracted twice by 1,2-dichloroethane-methanol-acetone (2:1:1). Constant shaking for 1 h with 200 mL solvent resulted an approximate yield of 7.63% (20). Leblebici et al. (26) also used Soxhlet method to extract cardamom seed oil using 5-7 g sample, 200 mL ethanol, and 7 h, which resulted a yield of 88.64 mg/g sample. The standard Soxhlet extraction (24 h) using *n*-hexane (200 mL; 2 g 35 mesh size particle green cardamom) was carried out at 69°C (22). The shake flask-solvent method with constant rotary shaking (190 rpm) using ethanol was also used to extract cardamom seed oil with approximate yield of 40 mg/g dry seed (40). Cardamom seed oil extraction was carried out using petroleum ether (Soxhlet extraction) for 24 h using w/v ration of 1:2 with 7.3% yield (48).

## Advance extraction techniques

For efficient extraction of bioactive phytochemicals and essential oils, there is a dire need to develop efficient, novel and robust methods since conventional methods contain many flaws. Regarding phytochemicals extraction from cardamom, advance techniques such as enzyme- assisted-, instant controlled pressure drop-, microwave- assisted-, pressurized liquid-, solar energy- based-, sub- critical-, supercritical fluid-, and ultrasound-assisted extractions have been developed (2, 10, 20–25). In recent years, practice of employing advanced extraction techniques in combination with conventional procedures to isolate bioactive phytochemicals from black and/or green cardamom has surged. For instance, ultrasound assisted extraction (UAE) as a pretreatment was carried out with subsequent hydrodistillation. The mixture with a solid to water (1:12) ratio was subjected to ultrasonication as a pretreatment to extract cardamom oil from seeds. Ultrasound generator was equipped with 19 mm tip, and probe was directly immersed at an approximate depth of 5 mm into the oleaginous mixture. Process parameters were as follows; maximal power output was 10 and 20% [from 30 W for 15 min (technique 2) and 30 min (technique 3) and 60 W 10 min (technique 4) and 15 min (technique 5) respectively]. Process was carried out at room temperature with 27, 36, and 54 KJ for all respective extraction techniques. Immediately after UAE, the mixture was submitted to hydrodistillation (41).

Microwave-assisted extraction (MAE) approach was used to obtain oil from green cardamom (*Elettaria cardamomum* L.). To ensure equal distribution of microwaves, a rotating microwave diffuser was used, and temperature monitoring was carried out by shielded thermocouple (ATC-300). For extraction, 100 g sample was soaked in water and then placed in the multimode microwave reactor. The obtained essential oil yield ranges between 1 and 2.27% based on the moisture contents, extraction time and microwave power (52). Recently, MAE was performed using 100 g black cardamom in the flask along with 100 mL water using microwave variables in 80W coupled with the Clevenger apparatus. MAE lasted for 70 min compared to 4 h hydrodistillation resulted a yield of 3.35% vs. 3.0% (24).

Supercritical fluid extraction is an advance extraction method with potential benefits, including less sample degradation, efficient material transfer, low risk of thermal denaturation of bioactive compounds, no sample clean-up, and its eco-friendly nature. Besides, it’s an efficient extraction technique because of easy control on operative conditions to liquefy carbon dioxide as the supercritical fluid through optimization of time, temperature, and pressure conditions (1, 6). Recently, Abdullah et al. (2) obtained cardamom phytochemicals by SFE using 99.8% pure carbon dioxide as a solvent (supercritical fluid) at a constant temperature (30°C) and pressure (300 bar) for a period of 60 min. Sub-critical extraction (SE) is also an advance procedure to extract phytochemicals using propane as an extracting solvent (20). In this procedure, 45 g pulverized seeds packed were placed in the extractor and propane pumped up at 1-1.5 L/min flow rate using a membrane pump. The extraction was carried out at 2-5 MPa pressure and 298 K. These researcher have also extracted oil using super- and sub-critical carbon dioxide (20).

The pressurized liquid extraction (PLE) approach was used to extract lipid fraction of green cardamom using response surface methodology. The influences of temperature (90-150°C), flow rate (1-4 mL/min), and ethanol-water concentration (25-75 wt.%) on essential oil yield were examined. Response surface equation revealed that the maximum yield was predicted at 90°C, 4 mL/min flow rate, and 75 wt.% ethanol. The PLE yield was the highest when compared to Soxhlet or hydrodistillation extractions (22).

The enzyme pre-treatment helps in disrupting the integral structure of cell walls for an improved liberation of free bioactive molecules. This treatment is still unable to completely isolate and release molecules because of reduced aqueous solubility (53). The super- or sub-critical solvents do not easily penetrate into multiple layers of cell membrane/wall thus extracting only free bioactive particles (23). The critical factors supposed to be optimized for sequential extraction (enzymatic pre-treatment followed by SFE) involves temperature, pressure, co-solvent, enzyme type, pressure, and exposure time (15). Chandran et al. (21) reported enzyme assisted hydrodistillation (EAHD), 100 g cardamom seed powder was pretreated with lumicellulase (mixture of four enzymes) with varying incubation time and concentrations. Citric acid was used to adjust mixture pH in the range of 5-5.5. Two hours incubation with enzyme concentration of 4 mg/mL/100 g of the substrate followed by hydrodistillation was optimized that resulted a yield of 2.5%. This corresponds to a marked increase in α-terpinyl acetate from 38.91 to 48.60% in enzyme-treated samples as compared to untreated one (21). Baby and Ranganathan (54) also used EAHD, and green cardamom sample (100 g) with 10% moisture was treated with Protease, Viscozyme L., Celluclast 1.5 L., and Pectinex Ultra SP-L. These enzymes (0.5-2%) were dissolved in 10 mL water and uniformly sprinkled on cardamom seeds. The pH was adjusted by citric acid between 4 and 5.5, incubation time and temperature varied from 30 to 120 min and 45-60°C. Afterward, the treated samples were subjected to hydrodistillation. The maximum yield was obtained at pH 5, 50°C, and 90 min. The highest yield (7.83%) was obtained with Viscozyme followed by Pectinex, Celluclast, and Protease respectively (7.68, 7.40, and 7.23%). The hydrodistillation of untreated cardamom seeds resulted in an oil yield of 6.75% (54).

Recently, the instant controlled pressure drop (DIC) technique as a pre-treatment before hydrodistillation to extract oil from green cardamom has also shown an increased oil yield of 4.40% (30 s, 140°C, 0.36 MPa) compared to 2.52% for control treatment (25). Moreover, a recent study also reported the potential of an eco-friendly, green and clean solar energy-based extraction (SEE) technique to extract green cardamom essential oil. SEE when compared to traditional hydrodistillation, extraction time was increased by 0.45 h with an optimized solar radiation of about 1000 W/m^2^. Further analysis revealed that SEE was 23-34% greener than the traditional hydrodistillation based on plant material type (9).

Conclusively, different extracting solvents were used to extract phytochemicals from different parts of black and green cardamom. The yield percentage varied depending upon extraction approach (intensity/power), extraction solvent, extraction time, raw material (plant parts), and moisture content of raw material, *etc*. Regarding the comparison of extraction time, HD and solvent extraction generally require 3-8 h, whereas Soxhlet needs even more time. The most abundantly used traditional extraction approach is HD followed by solvent or Soxhlet extraction (Table 2). The maximum oil fraction is supposed to be contained in the seeds of black and green cardamom. The maximum yield obtained from black cardamom was 3.71% from seeds using the HD approach (47). Regarding green cardamom, the maximum oil percentage (9.5%) was obtained from seeds using the HD approach (49). Leblebici et al. (26) obtained 8.8% oil from green cardamom fruits by Soxhlet extraction using ethanol as an extracting solvent. The solvents like petroleum ether, ethanol, and dichloromethane-acetone-methanol mixture seemed to be a promising option. The seeds in comparison to leaves, pericarp, whole pods/fruit, *etc*. contained maximum oil contents. Advanced extraction methods like MAE, EAE, SFE, PLE, or DIC have shown promising niches to be used as possible future strategies for extraction of phytochemicals from plant material. Moreover, the quality of cardamom phytochemicals extracted using greener (advanced) techniques was found superior in comparison to traditional methods.

**TABLE 2:** Comparative analysis of black and green cardamom: plant part, oil yield (%), extraction method, and extraction solvent.

Cardamom type	Plant part	Oil yield (%)	Extraction technique	Extracting solvent	References
**Black cardamom**	Fruit	3.5	SD^1^	Steam	(27)
	Seed	2.5	HD^2^	Water	(30)
	Seed	1.6, 2.7, 2.4	SD^1^	Steam	(31)
	Pericarp (husk)	0.18	HD^2^	Water	(32)
	Fruit	1.80	HD^2^	Water	(34)
	Seed	4.5	HD^2^	Water	(36)
	Rind	1.0	HD^2^	Water	
	Seed	0.98–1.95	HD^2^	Water	(37)
	Seed	1.2–2.8	HD^2^	Water	(43)
	Leaves	0.73	HD^2^	Water	(44)
	Seed	Not mentioned	HD^2^	Water	(45)
	Whole Pods (Chinese black cardamom)	0.7–1.8	HD^2^	Water	(46)
	Whole Pods (Indian black cardamom)	0.9–1.5	HD^2^	Water	
	Seed	3.71	HD^2^	Water	(47)
	Seed	3.3	MAHD^3^	Water	(24)
		3.0	HD^2^	Water	
**Green cardamom**	Fruit	2.5	SD^1^	Steam	(28)
	Capsules	3.68	SDE^4^	Dichloromethane	(29)
	Seed	5.5	SFE^5^	CO_2_	(16)
		5.0	HD^2^	Water	
		7.6	SE^6^	Hexane	
	Seed	6.0	HD^2^	Water	(33)
	Fruit coat	1.4	HD^2^	Water	
	Seed	Not mentioned	HD^2^	Water	(52)
		1-2.27	MAE^7^	Not applicable	
	Seed	5.4–6.6	SFE^5^	CO_2_	(20)
		6.8–7.2	Sub-critical	Propane	
		5.2–7.6	SE^6^	Dichloroethan–acetone–methanol	
	Seed	7.9–8.79	HD^2^	Water	(35)
	Fruit	7.7	SFE^5^	CO_2_	(26)
		6.6	HD^2^	Water	
		8.8	Soxhlet	Ethanol	
	Seed	1.9	HD^2^	Water	(21)
		2.5	EAHD^8^	Water	
	Seed	3.8	HD^2^	Water	(22)
		2.5	Soxhlet	*n*-hexane	
		11 (predicted)	PLE^9^	75 wt% ethanol	
	Fruit	3.1	HD^2^	Water	(39)
	Seed	4.5	HD^2^	Water	(40)
		Not mentioned	SD^1^	Water	
		4.0	SE^6^	Ethanol	
		Not mentioned	SFE^5^	CO_2_	
		Not mentioned	Subcritical	CO_2_	
		Not mentioned	Liquid extraction	CO_2_	
	Seed	7.03	HD^2^	Water	(41)
		6.9–7.4	UAE-HD^10^	Water	
	Seed	5.7	HD^2^	Water	(42)
	Seed	6.7	HD^2^	Water	(54)
		7.8	EAHD^8^		
	Fruit	2.6	HD^2^	Water	(48)
		7.3	SE^6^	Petroleum ether	
	Seed	Not mentioned	HD^2^	water	(45)
	Pod	5.2	HD^2^	water	(46)
	Seed	4.5–9.5	HD^2^	water	(49)
	Seed	2.52	HD^2^	water	(25)
		4.4	DIC^11^	Steam	
	Seed	4.1	HD^2^	Water	(9)
		4.4	SEE^12^	Not applicable	

^1^Steam distillation, ^2^hydrodistillation, ^3^microwave-assisted hydrodistillation, ^4^simultaneous distillation extraction, ^5^supercritical fluid extraction, ^6^solvent extraction, ^7^microwave-assisted extraction, ^8^enzyme-assisted hydrodistillation, ^9^pressurized liquid extraction, ^10^ultrasound-assisted hydrodistillation, ^11^controlled pressure-drop, ^12^solar energy-based extraction.

## Identification techniques

### Characterization and quantification of phytochemicals

The characterization and quantification of black and green cardamom-derived phytochemicals varies depending upon genotype, growing area, extraction method, moisture content, maturity levels, and identification or quantification protocol. To date, high-performance liquid chromatography coupled with photodiode array detector, gas chromatography (GC), GC-mass spectrometry (GC-MS), GC-flame ionization detection (GC-FID), and liquid chromatography-electrospray ionization quadrupole time-of-flight mass spectrometry have been used to characterize and quantify bioactive compounds present in phytochemicals (2, 6, 55). The following section presents reference studies focusing on the identification of cardamom-derived phytochemicals.

**FIGURE 2:**
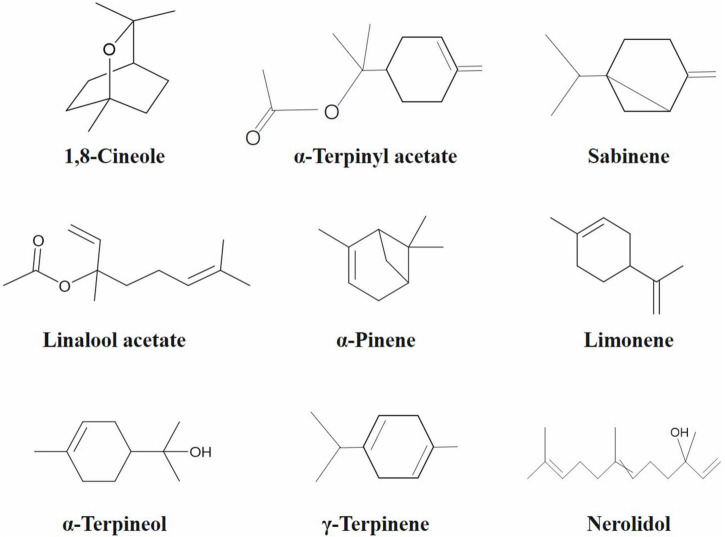
Chemical structures of major bioactive phytochemicals identified in cardamom.

Among chromatographic techniques, GC-MS is an advanced analytical technique widely applied for quantification of bioactive compounds present in cardamom phytochemicals (Figure 2 and Table 3). The GC-MS analysis of green cardamom-derived sample showed that it contains α-terpinyl acetate (34.95%), 1,8-cineole (25.30%), linalool acetate (8.13%), sabinene (5.48%), limonene (2.80%), α-terpineol (2.79%), α-pinene (1.81%), myrcene (1.76%), nerolidol (1.57%), *cis*-sabinene hydrate acetate (1.02%), geranyl acetate (1.02%), *n*-hexadecanoic acid (0.79%), α-farnesene (0.54%), geranial (0.45%), β-pinene (0.36%), geraniol (0.24%), α-thujene (0.20%), linalool oxide (0.15%), *p*-cymene (0.14%), and *g*-elemene (0.11%) phytochemicals (3). Likewise, GC-MS quantification of black cardamom sample obtained by SFE demonstrated 1,8-cineole (44.24%), α-terpinyl acetate (12.25%), nerolidol (6.03%), sabinene (5.96%), *g*-terpinene (4.30%), α-pinene (3.41%), methyl linoleate (3.11%), α-terpineol (2.85%), β-pinene (2.82%), *n*-hexadecanoic acid (2.74%), and limonene (1.02%), were the bioactive compounds along with some others present in minor quantity (2). The difference in the chemical composition of these two cardamom cultivars may be due to several factors, including genetics, origin, growth-, storage-, processing-, and experimental conditions.

**TABLE 3:** Major bioactive compounds identified in green and black cardamom by GC-MS (2, 3).

No.	Bioactive compounds	Green cardamom	Black cardamom
1	1,8-Cineole	25.30%	44.24%
2	*a*-Terpinyl acetate	34.95%	12.25%
3	Sabinene	5.48%	5.96%
4	Linalool acetate	8.13%	1.23%
5	α-Pinene	1.81%	3.41%
6	b-Pinene	0.36%	2.82%
7	Limonene	2.80%	1.02%
8	α-Terpineol	2.79%	2.85%
9	*g*-Terpinene	0.12%	4.30%
10	Nerolidol	1.57%	6.03%

The seed and rind of black cardamom of Nepal origin were hydrodistilled and obtained essential oils characterized using GC and GC-MS. Both oils contained 87 compounds making up 99.1 and 99% of the oils with 1,8-cineole, α-terpineol, β-pinene, and α-pinene as predominant monoterpenoids. Their respective concentrations were 60.8, 39.0, 9.8, and 8.3% and 17.7, 12.3, 6.4, and 4.8% in seed and rind essential oils (36). Joshi et al. (37) characterized black cardamom essential oil grown at various altitudes in Indian region (Himachal Pradesh) using GC-MS and GC-olfactometry (GC-O). Chemical profiling revealed 55 compounds making up 98% of essential oil. Predominant constituents include 1,8-cineole, nerolidol, β-myrcene, α-terpineol, α-terpinene, δ-3-carene, 4-terpineol, germacrene D, δ-terpineol, _*DL*_-limonene, and longifolenaldehyde. Out of 35 compounds responsible for aroma, 34 were identified using aroma extract dilution analysis. The most intense flavoring constituents were α-pinene, α-basabolol, _*DL*_-limonene, β-myrcene, and 1,8-cineole. They also reported presence of α-terpinyl acetate, a principal constituent of green cardamom in black cardamom (37).

Phytochemical profiling of green cardamom essential oil of Italian origin was accomplished by GC-MS, which identified 43 constituents. The compounds with significant concentrations were linalool (5.4%), linalyl acetate (8.2%), α-terpinyl acetate (42.6%), limonene (5.6%), and 1,8-cineole (21.4%) (16). Green cardamom seed- and fruit coat essential oils were characterized by GC-FID and GC-MS, which confirmed 25 constituents accounting for 95.28 and 96.58% of the seed- and fruit coat oils, respectively (33). The predominant phytochemicals in seed- and fruit coat oils include α-terpinyl acetate (56.87% vs. 51.25%), 1,8-cineole (15.13% vs. 23.74%), α-terpineol (4.67% vs. 5.25%), and limonene (4.05% vs. 3.82%), respectively (33). Green cardamom was also reported to have linalool, terpin-4-ol, α-terpineol, linalyl acetate, α-terpinyl acetate, and 1,8-cineole as predominant essential oil constituents (52). Chandran et al. (21) used GC-MS and GC-FID to characterize and quantify green cardamom essential oil constituents. Out of 18 identified compounds, α-terpinyl acetate and 1,8-cineole found in maximum concentration (48.6 and 32.8%). The green cardamom essential oil of Turkish origin was also quantified by GC-MS and GC-FID. Qualitative and quantitative analysis recognized 67 compounds accounting for 96.9% of the oil with 6.3% linalool, 40.7% α-terpinyl acetate, and 25.6% 1,8-cineole Savan and Kuçukbay (56).

Most recently, synthetic receptors with capability of specific molecular recognition which retain a targeted compound were designed namely molecular imprinting polymers or molecularly imprinted polymer (MIP_*S*_) (57). Debabhuti et al. (58) developed a sensor called quartz crystal microbalance (QCM) to detect β-pinene in cardamom essential oil using olive oil. It was suggested that Ω-9 and oleic acid found in olive oil had tendency to bind β-pinene. The developed sensor had shown notable selectivity for β-pinene as compared to other volatile constituents in cardamom. Similarly, QCM coated with MIPs having polymethacrylic acid was tailor-made to detect α-terpinyl acetate from cardamom. It was demonstrated that the sensor was extremely selective and sensitive toward α-terpinyl acetate detection (58).

## Therapeutic perspectives

### Antioxidant potential

In recent years, the role of oxidative stress on the pathophysiology of chronic diseases, including Alzheimer’s disease, cardiovascular disparities (atherosclerosis), cancer, diabetes, hypercholesterolemia, hypertension, immune deficiency, and Parkinson’s disease has been conferred. Briefly, the cellular metabolism of oxygen produces reactive oxygen species prompting an imbalance (between antioxidants and pro-oxidants), and subsequently developing an oxidative stress. Resultantly, an excessive production of reactive oxygen species had a negative effect on the cell aging and other cellular processes by a direct macromolecular damage or negatively affecting the regulation of cellular signaling pathways (2, 59–61).

**FIGURE 3:**
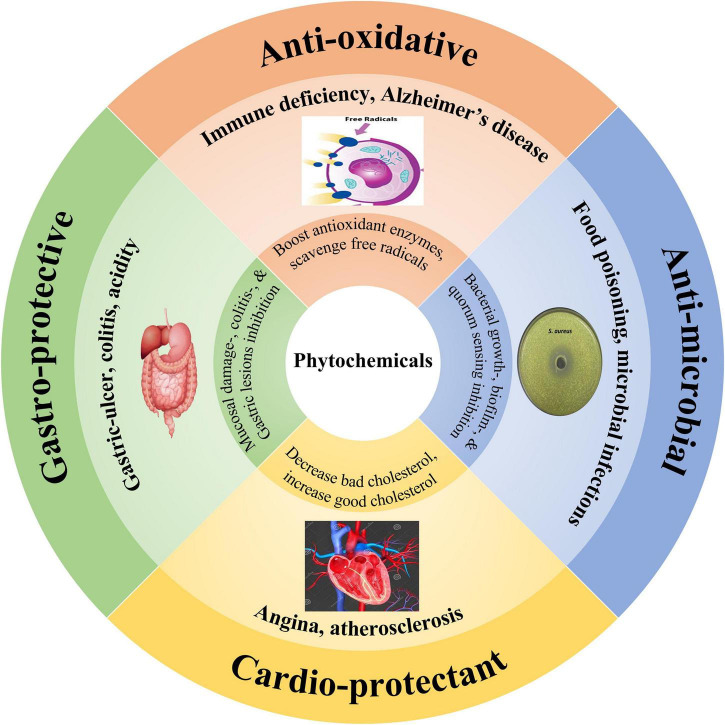
Therapeutic applications of cardamom phytochemicals, including anti-oxidative (cell image indicating free radicals deterioration vs. protection by the antioxidants), anti-microbial (petri-plate showing bacterial growth inhibition), cardio-protective (heart and arteries elaborating their beneficial effects), and gastro-protective properties (digestive system symbol signifying their stomach health-promoting activities).

Antioxidants can protect the biological systems against deteriorative oxidative processes by scavenging free radicals and help in the prevention and cure of physiological disorders. These functional substances are usually phytochemicals (polyphenols) which mainly include phenolics, phytosterols, tocols, and carotenoids (5). Research studies have reported that cardamom-derived phytochemicals proved helpful in boosting the antioxidant defense mechanism due to the presence of phenolics (kaempferol, quercetin, luteolin, and pelargonidin), phytosterols, and tocopherols (4). The antioxidants found in phytochemicals can scavenge and inactivate free radicals, offering protection to cells against deleterious effects of oxidative processes (Figure 3). Besides, cardamom-derived phytochemicals can significantly enhance glutathione and superoxide dismutase (antioxidant enzymes) and decrease malondialdehyde levels in the body (6).

Chowdhury and Islam (62) evaluated the anti-oxidative properties of methanolic extract of green cardamom leaves by measuring scavenging ability of 2,2 Diphenyl-1-picrylhydrazyle (DPPH) free radicals responsible for oxidative reactions. In this study, the IC_50_ value observed with DPPH assay against leaves extract was 594.47 μg/mL, indicating remarkable anti-oxidative potential due to substantial presence of flavonoids, phenols, saponins, terpenoids, and triterpenes phytochemicals. Saeed et al. (63) reported the anti-oxidative effects of aqueous and methanolic extracts obtained from green cardamom seeds and pods. Purposely, total phenolic compounds and antioxidant activity were determined by the Folin–Ciocalteu phenol reagent, DPPH and linoleic acid peroxidation inhibition methods. The results indicated that total phenolic compounds ranges 27.75-126.35 mg gallic acid equivalent (GAE)/g dry weight, DPPH scavenging ability 46-91% with the extract concentration of 5 mg/L and linoleic acid peroxidation inhibition of 34-83%.

Ramadan et al. (4) determined total phenolic compounds in cardamom and olive oils by Folin-Ciocalteu reaction, and reported that cardamom had 3.9 ± 0.02 mg GAE/g oil, higher than olive oil 3.5 ± 0.01 mg GAE/g oil. Furthermore, cardamom phytochemicals antiradical activity was evaluated by DPPH⋅ and galvinoxyl stable free radicals assays. DPPH assay showed that cardamom had promising radical scavenging activity; after 30 min and 60 min of incubation, 26 and 32% free radicals were scavenged by cardamom phytochemicals. Moreover, antiradical activity tested by electron spin resonance spectrometer apparatus showed that cardamom phytochemicals quenched 22 and 33% free radicals after 30 and 60 min of incubation with galvinoxyl radicals, respectively. The authors reported remarkable antioxidative- and antiradical properties were due to phytochemicals such as tocopherols, phenolic compounds and phytosterols found in cardamom.

### Antimicrobial potential

Foodborne pathogens and especially microbial recurrent infections occurred due to biofilm formation have attracted attention of researchers, dietitians and chemists to explore organic bioactive compounds as safe alternative to conventional antibiotics. “Biofilm is a three-dimensional structure produced by mono- or multi-microbial communities embedded into an excreted extracellular matrix providing protection/resistance to pathogens against the immune systems, antimicrobials, and harsh environmental conditions.” The process of bacterial biofilm formation involves five stages, including microbial adhesion by bacterial flagella/pili, exopolysaccharide secretion, spatial three-dimensional structure formation, maturation of biofilm, and cellular detachment to expand the microbial community. This process of biofilm formation is governed by bacterial communication system called quorum sensing that plays role in the synthesis, release and detection of signaling molecules (2, 64, 65). Thus, inhibition of quorum sensing is crucial to inhibit biofilm formation to eradicate persistent infections as bacteria causing different hospital-acquired infections (60-70%) and human infections (∼80%) had revealed biofilm-origin (66).

Cardamom-derived phytochemicals exhibited antimicrobial properties and effectively inhibited the growth of pathogenic microorganisms i.e., bacterial growth-, biofilm formation- and quorum sensing inhibition ascribed to alkaloids, flavonoids, phenolics, tocols, terpenes, tannins, sterols, steroids, and saponins (2, 3, 8). Numerous studies have reported the growth inhibitory activities against Gram-negative and Gram-positive bacteria and foodborne fungi. Ramadan et al. (4) reported that cardamom phytochemicals exhibited antimicrobial activities against dermatophytic fungi (i.e., *Trichophyton mentagrophytes* and *Trichophyton rubrum*), and foodborne pathogens such as *Escherichia coli* ATCC 25922, *Listeria monocytogenes* ATCC 15313, *Staphylococcus aureus* ATCC 8095, and *Salmonella enteritidis* ATCC 13076. Briefly, antimicrobial properties measured against pathogens in terms of clear zones diameter were 32, 30, 17, 15, 18, and 20 mm for *T. mentagrophytes, T. rubrum*, *S. aureus*, *E. coli*, *S. enteritidis*, and *L. monocytogenes*, respectively.

Abdullah et al. (3) reported that green cardamom-derived phytochemicals extracted by SFE effectively inhibited *Escherichia coli* O157:H7 and *Salmonella* Typhimurium JSG 1748 biofilm formation by 64.29, 65.98, 70.41, and 85.59%, and 6.13, 45.50, 49.45, and 100%, respectively at 0.015, 0.031, 0.062, and 0.125% (v/v) concentrations, respectively. In regards to quorum sensing inhibition, the 0.031 and 0.062% concentrations inhibited the production of violacein (an indicator of inhibition/enhancement) without significant impact on growth of *Chromobacterium violaceum* ATCC 12472. Likewise, black cardamom-derived phytochemicals obtained through SFE prevented *Escherichia coli* O157:H7 and *Salmonella* Typhimurium JSG 1748 biofilm formation by 47.31, 54.15, 76.57, 83.36, and 84.63%, and 33.67, 34.14, 38.66, 46.65, and 50.17%, respectively at 0.031, 0.062, 0.125, 0.25, and 0.5% (v/v) concentrations (2). Furthermore, a concentration of 0.5% suppressed production of violacein without affecting growth of *Chromobacterium violaceum* ATCC 12472, whereas 1% concentration resulted in complete inhibition of violacein (100%) along with growth inhibition (30%). Alam et al. (67) reported that aqueous and ethanol extracts of green cardamom at concentrations of 200 and 100 mg/mL revealed anti-inflammatory potentials. Shukla et al. (68) indicated that methanolic and ethyl extracts of black cardamom at dose of 100-300 mg/kg and 200-400 mg/kg presented significant analgesic effects.

### Cardio-protectant and hypo-cholesterolemic potential

Worldwide, unhealthy-lifestyle, poor and imbalanced dietary habits are the leading causes of chronic maladies, including atherosclerosis, hypercholesterolemia, and hypertension. Cardiovascular disparities may occur due to high levels of bad-cholesterol and sugar in blood, inflammation of blood vessels, increased platelets aggregation, and smoking (6, 69). Furthermore, oxidation of low-density lipoproteins initiates the endothelial dysfunction leading to the formation of complex-plaques and causing substantial resistance to arterial blood flow. When these plaques rupture, the platelet cells stick on the injury site and form clumps and subsequently develop blood clots. These blood clots can narrow the coronary arteries and lead to the progression of angina, and ultimately cause heart attacks upon worsening of angina (70, 71). For a healthy person, the total cholesterol should be less than 200 mg/dl, including high-density lipoproteins greater than 40 mg/dl in men and 50 mg/dl in women, while low-density lipoproteins less than 130 mg/dl (72).

Lahlou et al. (73) conducted an animal study to investigate the cardiovascular function of 1,8-cineole, a main bioactive phytochemical found in cardamom. The study’s results showed that intravenous bolus injections of 1,8-cineole at concentrations of 0.3-10 mg/kg caused reduction in aortic pulse pressure in a dose-dependent manner. Goyal et al. (74) reported that experimental rats treated with cardamom for a period of 30 days showed high antioxidants contents and observed less heart damage after a heart attack due to antiradical and anti-oxidative activities. Furthermore, cardamom supplementation presented cardio-protective properties against isoproterenol-induced myocardial infarction by mitigating left ventricular and hemodynamic weakness, and augmenting antioxidant defense led to the preservation of cardiomyocytes demonstrated by decreased leakage of myocytes injury marker enzymes. The aforementioned facts suggest that cardamom had potential as a cardio-protectant to attenuate oxidative stress-mediated cardiovascular and heart damages or dysfunctions attributed to phenolics (phenolic acids and flavonoids) and sterols (74, 75).

Nagashree et al. (12) investigated the hypocholesterolemic effects of cardamom phytochemicals in Wistar rats and reported that oral administration significantly decreased total-, low- density-, very low-density lipoproteins, and triglycerides levels contributing cardiovascular diseases. Abdullah (6) conducted a bio-evaluation study on Sprague Dawley rats, and investigated the hypocholesterolemic effects of encapsulated essential oils of green and black cardamom. Interestingly, the encapsulated oils exhibited significant affects in reducing body weights of experimental rats, however, black cardamom caused maximum reduction in body weight. Briefly, black cardamom phytochemicals caused reduction in total cholesterol by 12.53% and low-density lipoproteins by 14.08%. Furthermore, this intervention improved the serum antioxidant status since glutathione contents increased and thiobarbituric acid reactive substances decreased. Conclusively, phytochemicals imparted beneficial effects by boosting serum antioxidant enzymes, increasing high-density lipoproteins and substantially decreasing triacylglycerides, total cholesterol, phospholipids, low-density lipoproteins, and very low-density lipoproteins levels (6, 12, 75).

## Gastro-protective properties

Among gastrointestinal illnesses, gastric-ulcer is one of the chronic gastrointestinal disease. In recent years, extensive efforts have been made to explore phytochemicals as nutraceuticals to encounter gastrointestinal disorders with a special focus on gastric-ulcer. In this regard, herbaceous plants such as ginger and cardamom derived phytochemicals have reported their remarkable potentials with promising gastroprotective activities. For instance, 1,8-cineole a major phytochemical found in cardamom had gastro-protective properties and helped in the prevention and cure of colitis and gastric injuries (Figure 3). Santos and Rao (76) conducted a bio-evaluation study in which rats were fed on 1,8-cineole at a concentration of 50-200 mg/kg, an hour prior to the consumption of 1 mL of pure ethanol. The study results indicated that 1,8-cineole exhibited gastro-protective effects against ethanol-induced mucosal damage and significant reduction was observed in ethanol-induced gastric injury. In another study, Santos et al. (77) described that rats pretreated with 1,8-cineole imparted gastro-protective effects by causing significant reduction to gross damage scores and wet weights (mg/cm) of colonic segments in trinitrobenzene sulfonic acid-induced colitis.

Several other studies have also reported the gastro-protective properties of cardamom especially its main bioactive constituent 1,8-cineole. For example, different cardamom-derived phytochemicals such as methanolic extract (100-500 mg/kg), essential oil (12.5-50 mg/kg), petroleum ether soluble (12.5-150 mg/kg), and insoluble fractions (450 mg/kg) of the methanol extract have effectively inhibited gastric lesions in rats induced by aspirin and ethanol. These cardamom phytochemicals offered gastro-protective effects through substantial inhibition of gastric lesions attributed to antioxidative, antiradical and antiinflammatory activities of bioactive constituents (13). Bhaswant et al. (78) described that male Wistar rats were fed on trans-fats for 16 weeks to induce metabolic syndrome, and diet supplementation of 3% dried black cardamom for the last 8 weeks effectively reversed the signs of metabolic syndrome. Besides, black cardamom supplementation also increased the amount of complex carbohydrates in the body which significantly improved the gastrointestinal functions because dietary fiber attenuates obesity (79).

## Biosafety and non-mutagenic properties

The U.S. Food and Drug Administration does not strictly regulate the phytochemicals (herbs and spices) applications as they were applied as biomedicine to treat ailments since ancient times. The cardamom-derived phytochemicals use for pharmaceutical and food applications is suggested as a safe alternative to conventional anti-infective agents and chemical preservatives because of its proved biosafety on the human health. Recently, the non-mutagenic activity and biosafety of green cardamom-derived phytochemicals have been confirmed through Ames test (3, 80). A phytochemical can be appraised mutagenic only “if after the incubation period of Ames test, the number of positive-wells (produce yellow, partial yellow, or turbid colors) with the phytochemical are higher than twice of positive-wells with negative control plate (standard mutagens being used to induce mutation, e.g., potassium dichromate and sodium azide)” (81). Abdullah et al. (3) investigated the mutagenic activity of green cardamom-derived phytochemicals extracted via SFE. It has been observed that tested samples had non-mutagenic activity because they did not cause mutation in *Salmonella* Typhimurium TA98 and *Salmonella* Typhimurium TA100, selected as representative mutant bacterial strains. Previously, Saeed et al. (63) also investigated the mutagenic potential of aqueous and methanolic extracts obtained from green cardamom which exhibited strong non-mutagenic potential toward mutant strains *Salmonella* Typhimurium TA98 and *Salmonella* Typhimurium TA100.

## Encapsulation and delivery strategies

**FIGURE 4:**
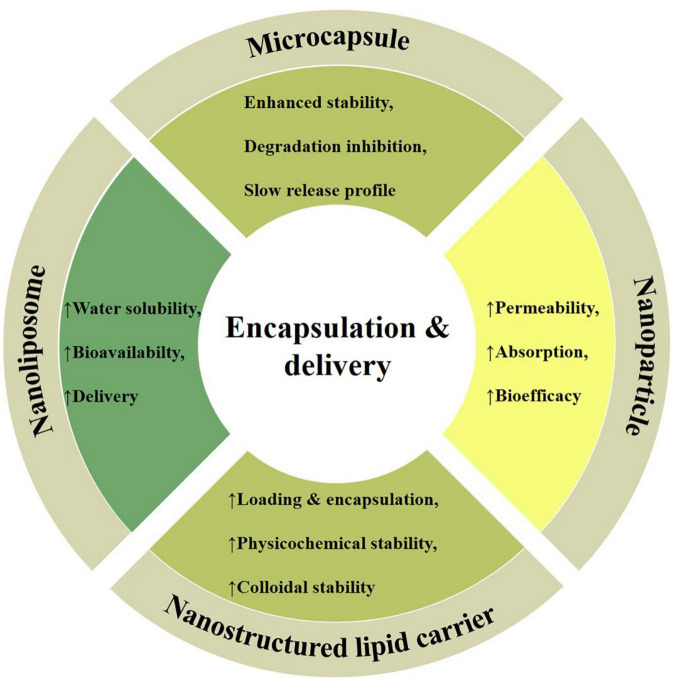
Different encapsulation and delivery strategies, including; microcapsules (enhanced stability and controlled release), nanoparticles (higher absorption and bioactivity), nanostructured lipid carriers (improved physicochemical stability), and nanoliposomes (higher solubility and bioavailability) with their unique properties being employed as efficient carriers of cardamom phytochemicals.

Phytochemicals low solubility in aqueous medium restrict their applications in food and pharmaceutical products, thus, encapsulation is a tool to enhance their stability, bioavailability and bio-efficacy by protecting them against adverse environmental conditions, improving retention time and controlled release at target sites (82, 83). In this regard, delivery techniques and wall materials are the key strategies enabling dietary phytochemicals applications as effective biotherapeutic agents against various physiological disorders and pathogens. The different types of wall materials being used in the encapsulation include proteins (casein, whey, and zein), carbohydrates (starches, dextrin and sucrose), gums (sodium alginate, agar, gum arabic, and guar gum), and lipids (waxes, hardened oils and fats) (6, 84, 85). The significance of encapsulation and efficient delivery vehicles in terms of carriers of cardamom phytochemicals (e.g., microcapsules, nanoparticles, nanostructured lipid carriers, and nanoliposomes) in improving the physical stability as well as preserving the antioxidant and antimicrobial properties are reviewed in the following section (Figure 4).

## Microcapsules

“Microcapsule is a hollow reservoir system consists of a core and a wall (matrix shell) encapsulating bioactive substances, and the average particle size varies from 1 to 1000 μm.” Microcapsule application as a delivery vehicle possesses attractive advantages such as enhanced stability, degradation inhibition, transportation convenience, slow release characterization, and targeted efficient delivery as well as protection against the harsh external environment to the bioactive molecules. These characteristics making them potential delivery vehicles for food, cosmetic, nutraceutical, and pharmaceutical applications (86).

Zandi et al. (87) fabricated microcapsules comprising alginate and whey protein concentrates containing cardamom phytochemicals through emulsification/internal gelation technique. The formed microcapsules had particle size of 15–110 μm and high encapsulation efficiency of 83.67%. The study’s findings demonstrated that the release from the microcapsules was through the classical Fickian diffusion mechanism at all conditions, including pH, release media temperature and shear force. The authors suggested that microcapsules not only protected the encapsulated cardamom essential oil for a longer time in the aqueous phase; but also presented better release profile at high temperatures. Mehyar et al. (88) produced freeze-dried microcapsules from emulsions constituting (*w/w*): whey protein isolate (15 or 30%); guar gum (0.1%), and carrageen (0.2%) as wall material, and cardamom essential oil as core material (10% of biopolymers concentration). The obtained cardamom essential oil-loaded microcapsules comprising 30% whey protein isolate were found most effective in entrapping oil and exhibited the highest microencapsulation efficiency (98.56%) due to good emulsification properties of whey protein isolates and their ability to produce thicker and less porous microcapsules. Moreover, whey protein isolates and whey protein isolates:guar gum formulations were effective in retaining the flavor volatile compounds such as 1,8-cineole and D-limonene (the *t*_1/2_ of 1,8-cineole and D-limonene increased by 15.36 and 13.03 weeks) during 16 weeks storage at 20°C and 35°C. The addition of guar gum increased the surface porosity and decreased shape regularity, therefore, 30% whey protein isolates was the best formulation as wall material in improving the stability and flavor retention (88). Likewise, Krishnan et al. (84) reported that microencapsulation of cardamom oleoresin in ternary complexes (gum arabic/maltodextrin/modified starch: 74.50%:12.75%:12.75%) by spray drying significantly enhanced the storage stability and flavor retention properties. During storage at 25°C, the *t*_1/2_ of 1,8-cineole, α-terpinyl acetate and volatiles increased from 27.72, 60.78, and 47.79 weeks to 231, 223.54, and 173.25 weeks compared to non-encapsulated cardamom oleoresin (84).

## Nanoparticles

“A nanoparticle is a nano entity with all the dimensions and structures in the nanoscale range (1-1000 nm).” In recent years, applications of nanoparticles have emerged as efficient delivery vehicles due to their properties including, high surface to volume ratio, ultra-small sized dimensions, easy penetration and absorption, and bioactivity enhancement (89).

Ouerghi et al. (90) designed green cardamom essential oil loaded titanium dioxide nanoparticles prepared by the classical sol-gel method with a hydrodynamic diameter of 335.6 nm. The obtained nanoparticles exhibited significant antibacterial activities against Gram-positive (*Bacillus subtilis*), and Gram-negative bacteria (*Escherichia coli*) with a minimum inhibitory concentration (MIC) of 18.75 μg/mL. Furthermore, the results demonstrated that essential oil loaded nanoparticles exhibited better bactericidal effects against human pathogenic bacterial strains compared to non-encapsulated cardamom essential oil (18.75 μg/mL vs. 25 μg/mL), demonstrating efficiency of the novel biotherapeutic agent. Likewise, Singh et al. (91) synthesized black cardamom oil loaded silver nanoparticles (20-30 nm) via a simple one step mechanism (oxidation of 1,8-cineole and reduction of silver ions). It has been reported that size and shape of the nanoparticles could be tuned by varying concentration of black cardamom oil. Briefly, a lower concentration produced spherical and monodispersed nanoparticles of smaller-sized, whereas, a higher concentration produced larger-sized and polydispersed particles because of slower and faster reactions, respectively. Moreover, a thin film comprising silver nanoparticles and gelatin (composite film) was prepared and its antibacterial effectiveness was tested against Gram-positive and Gram-negative bacteria. The results revealed a complete growth inhibition in the case *Escherichia coli* and *Salmonella* Typhimurium (Gram-negative bacteria) at a concentration of 100 μL, however, this concentration could not completely suppressed the growth of Gram-positive bacteria (*Streptococcus pneumonia* and *Staphylococcus aureus*). The aforementioned results have shown remarkable antibacterial properties of cardamom phytochemicals loaded nanoparticles which could be used in developing biotherapeutic agents such as organic antibiotics and films for industrial applications.

## Nanostructured lipid carriers

“Nanostructured lipid carrier is a spherical nanoparticle with a lipid core formed by the mixture of solid and liquid lipids surrounded by a lipophilic bilayer membrane that exhibits potential of incorporating both lipophilic and hydrophilic bioactive substances.” Nanostructured lipid carrier offers special advantages, including high loading and encapsulation efficiency, minimized expulsion, enhanced local deposition of bioactive compounds, higher colloidal stability due to higher density of solid lipids, and good physicochemical stability (less mobility of bioactive substance in its solid matrix) (92, 93). Therefore, rational design of nanostructured lipid carrier has great influence on the physical and functional properties as well as release behavior of encapsulated compounds.

Nahr et al. (94) developed cacao butter-based nanostructured lipid carrier containing cardamom essential oil via low energy emulsification equipped with high shear homogenization and sonication. The obtained nanostructured lipid carrier showed particle size of 118.7 ± 1.2 nm, entrapment efficiency more than 90%, and high storage stability (94). In addition, nanostructured lipid carrier remarkably preserved the antimicrobial property of cardamom essential oil (MIC = 1100 lg/mL toward *E. coli* and *S. aureus*) during a storage of 30 days compared to non-encapsulated oil (MIC = 2200 lg/mL and 4400 lg/mL against *E. coli* and *S. aureus*). Recently, Nahr et al. (93) fabricated cardamom essential oil-loaded round-shaped nanostructured lipid carrier using cocoa butter and olive oil, and formed nanoparticles size remained under 150 nm and entrapment efficiency greater than > 90%. *In vitro* release study revealed that 40-55% of cardamom essential oil was released from nanostructured lipid carriers in 40 days, and also protected its antioxidant potential compared with cardamom essential oil emulsion as 5.7 and 12.32% reduction was observed after 30 days, respectively (93). In summary, nanostructured lipid carriers are advanced generation delivery vehicles that could be used to control release, prolong residence time and enhance permeability to maximize the health-promoting benefits of phytochemicals.

## Nanoliposomes

“A liposome is a spherical structure formed by the interactions of phospholipids such as phosphatidylcholine with the plant-derived bioactive compounds in an appropriate solvent.” When a liposome is dispersed in an aqueous solution, the phospholipids immediately produce vesicles having bilayer membrane that work as carriers of hydrophobic and hydrophilic bioactive compounds. Moreover, bilayer membrane increases the water solubility of lipophilic compounds and also acts as a strong barrier against adverse environmental conditions (e.g., pH, oxygen, and light). When the size of liposome is in the nanoscale range (1-1000 nm), its water solubility enhances due to increased surface area of the particles produced in new dispersed phase, bioavailability increases because of efficient crossing from permeability barriers, and subsequently improved delivery (82, 95, 96).

Nahr et al. (96) fabricated nanoliposomes using lecithin to encapsulate cardamom essential oil via thin layer hydration method coupled with homogenization and sonication. Dynamic light scattering revealed that the obtained nanoliposomes had particle size less than 150 nm, and zeta potential values (–10.9 to –17.4 mV) remained constant during a storage of 30 days. Moreover, the cardamom essential oil-loaded nanoliposomes showed high encapsulation efficiency (>60%), remarkable physical stability even after 30 days and also protected antimicrobial and antioxidant activities. The authors suggested that cardamom essential oil-loaded nanoliposomes have potential as natural food preservative and antioxidant for a wide range of application in the food, cosmetic, nutrition, and pharmaceutical industries. Paul et al. (97) formulated biotherapeutic PEGylated nanoliposomes using soya phosphatidylcholine and Tween 80 by probe-sonication to protect and enhance the therapeutic potential of green cardamom essential oil to attenuate type 2 diabetes and hypercholesterolemia. The *in vitro* investigations of the resultant nanoliposomes showed an entrapment efficiency of 84% and significant antioxidant activity (DPPH scavenging potency was 59%) due to antioxidant substances present in cardamom phytochemical. Moreover, *in vivo* therapeutic bioefficacy study revealed that an oral administration of loaded liposomes to Wistar albino rats at a concentration of 550 mg/kg of body weight for 35 days successfully restored their normal fasting blood glucose and serum lipid profile levels by up-down regulations of the related key enzymes.

## Conclusion and future perspectives

Dietary phytochemicals (e.g., alkaloids, carotenoids, organosulfur compounds, phenolics, and phytosterols) are health-promoting bioactive moieties that help in the prevention and mitigation of physiological disorders and microbial infections. Researchers have designed and applied various extraction procedures, including conventional- (e.g., hydrodistillation, steam distillation and Soxhlet extraction) and advance extraction techniques (e.g., enzyme- assisted-, instant controlled pressure drop-, microwave- assisted-, pressurized liquid-, solar energy- based-, sub- critical-, supercritical fluid-, and ultrasound-assisted extractions) to extract cardamom phytochemicals. Different identification techniques such as GC, GC-MS, and GC-FID revealed that 1,8-cineole and α-terpinyl acetate were the principle bioactive constituents in black and green cardamom. Regarding therapeutic potential of cardamom phytochemicals, studies have shown their effective role in mitigating foodborne pathogens, oxidative stress, cardiovascular-, and gastrointestinal diseases. Encapsulation and delivery of phytochemicals through microcapsules, nanoparticles, nanostructured lipid carriers, and nanoliposomes were effective strategies with promising advantage, including increased stability, permeability, controlled release, bioavailability, and bioefficacy of embedded bioactive substances.

The inclusion of cardamom in food should be promoted due to its polyphenols profile which may help in improving the antimicrobial status, shelf life, and quality of products as well as alleviating oxidative stress and various lifestyle related disorders. The literature review indicated that cardamom phytochemicals had biotherapeutic potential, however, certain important aspects should be taken into consideration for future research. For example, the optimum dose of cardamom-derived bioactive compounds required to obtain health benefits, the physical form for supplementation to ensure maximum bioavailability at target sites, and potential health risks associated with any bioactive component, issues need thorough investigations.

## Author contributions

Abdullah and JX: conceptualization. Abdullah, NA, WT, and SZ: writing-original draft preparation. Abdullah, YZ, SF, QH, and JX: writing-review and editing. JX: supervision and funding acquisition. All authors read, revised, and agreed to manuscript publication.
